# Primary Carcinoma In Situ of the Ureter Without Concurrent Renal Pelvis or Bladder Carcinoma: A Case Report and Literature Review

**DOI:** 10.7759/cureus.43453

**Published:** 2023-08-14

**Authors:** Mohamed Farah, Wesam Al-Dhahir, Mosea Song, Wasim Mahmalji, Ahmed Mohamed

**Affiliations:** 1 Urology, Wye Valley NHS Trust, Hereford, GBR

**Keywords:** literature review, ureteroscopy, computed tomography, radical laparoscopic nephroureterectomy, primary ureteral carcinoma in situ

## Abstract

Primary carcinoma in situ (CIS) solely affecting the ureter without concurrent involvement of the kidney or bladder is an exceptionally rare condition. This case report presents the clinical management of a 67-year-old male patient with primary ureteral CIS, highlighting the diagnostic workup, surgical approach, and postoperative outcomes. The diagnosis was established through the use of CT and ureteroscopy, leading to the decision for radical laparoscopic nephroureterectomy. Additionally, a comprehensive literature review was conducted to discuss the diagnostic challenges and management options for primary ureteral CIS.

## Introduction

Upper urinary tract urothelial carcinoma (UTUC) is a relatively rare disease, accounting for only 5-10% of urothelial carcinoma, with an estimated annual incidence of 1-2 cases per 100,000 people per year in Western countries [1]. Concomitant upper tract urothelial carcinoma in situ (UT-CIS) is reported to be present in 11%-36% of patients with UTUC. Primary carcinoma in situ (CIS) that solely affects the ureter without concurrent involvement of the kidney or bladder is even rarer [1,2]. Herein, we describe a case of primary ureteral CIS without associated other urinary tract tumors, which was managed with a radical nephroureterectomy.

## Case presentation

**Figure 1:**
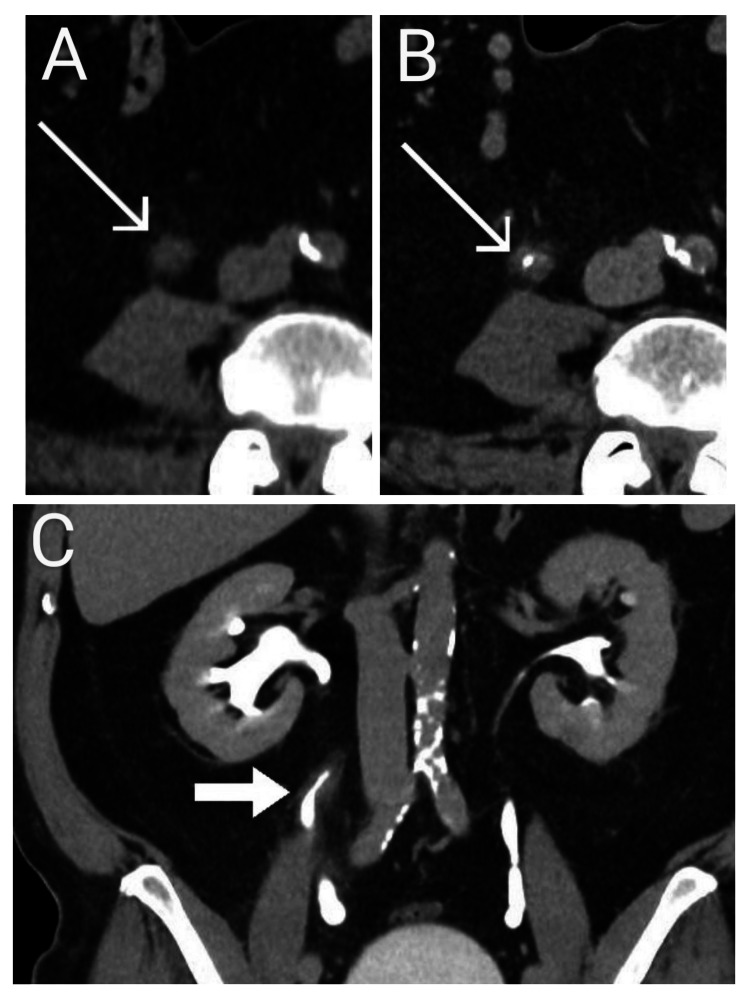
Standard venous phase (A), along with axial (B) and coronal (C) views in the excretory phase. CT showed diffuse thickening of the wall in the right mid-ureter, along with a region of relatively constricted lumen in the mid-ureter. There was no discrete mass or other collecting system filling defects detected.

A 67-year-old male ex-smoker with a history of hypertension presented with persistent flank pain and non-visible haematuria. An initial CT scan without contrast revealed inflammatory changes in the right ureter, suggestive of the passage of a urinary tract stone. Subsequent imaging three months later, with a CT urogram, demonstrated persistent inflammation and ureteral wall thickening in both the upper and mid portions of the right ureter (Figure 1). A flexible cystoscopy to assess the lower urinary tract was normal. A right-sided retrograde pyelogram confirmed the presence of narrowing in the mid-ureter, and ureteroscopy revealed abnormal mucosal induration in the upper and mid portions of the right ureter without an obvious tumor. The histology from the ureteral biopsies revealed inflamed subepithelial tissue with a collection of cells exhibiting high morphology, representing a focus of CIS. The ureteral biopsy results were discussed in a multi-disciplinary team meeting (MDT), leading to the decision to perform a radical nephroureterectomy. This decision aligned with the European Association of Urology (EAU) guidelines for UTUC, which recommend radical nephroureterectomy for managing localized high-risk disease. Kidney-sparing surgery, including ureteroscopy or segmental ureterectomy, was not considered due to the greater risk of progression and the direct impact on survival for high-risk UTUC, according to the EAU guidelines. A right-sided radical nephroureterectomy with excision of a bladder cuff was performed. The specimen showed no evidence of an intraluminal mass within the renal calyces, pelvis, or ureter. Further histological examination of the thickened ureter revealed extensive urothelial carcinoma in situ, noted at a 50 mm distance from the pelvic-ureteric junction. The distal 40 mm length of the ureter was sampled, and no convincing evidence of CIS or papillary transitional cell carcinoma was found. A check rigid cystoscopic examination and mapping biopsies of the bladder were carried out to ensure that a concurrent bladder malignancy had not been overlooked; however, no evidence of malignancy within the bladder was found. The patient is now undergoing surveillance of his lower urinary tract with check flexible cystoscopy every three months, including urine cytology and a six-monthly CT scan with contrast.

## Discussion

UT-CIS poses a diagnostic challenge due to its rarity and difficulty in detection; therefore, a multimodal diagnostic approach of urine cytology and imaging, such as CT/MR urography and ureteroscopy, is required to detect and diagnose UT-CIS. However, even with such an approach, detection of UT-CIS may require further tests. CT urogram has the highest diagnostic accuracy for UTUC, with a sensitivity of 92% and a specificity of 95% [3,4]. Early CIS and flat lesions without focal wall thickening are not detectable on CT urogram [1]. Although standard white light ureteroscopy is a valuable diagnostic tool, it is essential to note that it may have limitations in detecting CIS. A study of 300 patients evaluating the diagnostic reliability of prenephroureterectomy ureteroscopy for the detection of upper tract CIS found that the sensitivity of ureteroscopic biopsies for detecting CIS was low, with only 60% of patients with CIS on postnephroureterectomy histology having positive ureteroscopic biopsies [5]. However, further studies have shown that the use of additional techniques, such as photodynamic diagnosis (PDD) or narrow-band imaging (NBI), can improve the detection of CIS during ureteroscopy [6]. Zhang G et al. reported a case of CIS of the proximal ureter that was only detected using a fluorescence in-situ hybridization (FISH) of the urine. Overall, they reported that, in their case series, the urine FISH had a sensitivity of 84% and a specificity of 91%, compared to urine cytology’s sensitivity of 53% and specificity of 91% [7]. Radical nephroureterectomy is the gold standard treatment for UT-CIS, especially in patients with high-grade ipsilateral carcinoma [8]. In a multicenter study of 1363 patients who underwent radical nephroureterectomy conducted by Karam JA et al., only 2% (or 28 patients) had primary CIS alone in the final specimen, with a reported 84.2% three-year recurrence-free survival and an 88.8% three-year disease-specific survival [9]. Additionally, Yuasa T et al. followed up on eight patients who underwent radical nephroureterectomy (with a bladder cuff), reporting that all eight patients were alive at a mean follow-up time of 56 months [10]. Non-surgical treatment is receiving more attention, especially in patients who are not eligible for surgery, with a solitary functioning kidney or bilateral involvement. Bacillus Calmette-Guerin (BCG) therapy is a minimally invasive method for treating UT-CIS. In a prospective study of 17 patients with upper urinary tract CIS, Kojima Y et al. reported that BCG therapy was equally effective compared to nephroureterectomy. They reported a 91% five-year cancer-specific survival rate for those who received BCG therapy and 80% for those who underwent nephroureterectomies, with no significant difference between the two groups (p = 0.62) [11]. In addition, Horiguchi H et al. performed a retrospective study of 58 patients with UT-CIS, comparing the outcomes of BCG therapy and nephroureterectomy. They reported no significant difference in five-year survival rates between those who received BCG therapy and those who underwent nephroureterectomy [12].

Foerster B et al. conducted a meta-analysis to evaluate the oncologic outcomes of adjuvant endocavitary instillation following kidney-sparing surgery (KSS) for UTUC. The study included 27 eligible reports comprising 438 patients, with 18 studies used for quantitative analyses. The analysis found that adjuvant instillations in Ta-T1 patients resulted in a 40% upper tract recurrence rate, 94% cancer-specific survival, and 71% overall survival. For patients with UT-CIS treated with BCG, the cytology response rate was 84%, upper tract recurrence rate was 34%, and progression rate was 16%. The results did not show significant differences between instillation approaches. The authors concluded that endocavitary instillations in UTUC did not reveal any differences between regimens and approaches. However, the efficacy remains to be demonstrated, with the potential for novel drugs to impact treatment paradigms [13]. The EAU guidelines recommend considering kidney-sparing surgery, including ureteroscopy or segmental ureterectomy, on a case-by-case basis for high-risk patients with imperative indications such as solitary kidney, bilateral UTUC, chronic kidney disease, or any other comorbidity compromising the use of radical nephroureterectomy. However, there is a greater risk of progression after kidney-sparing surgery for high- vs. low-risk UTUC with a direct impact on survival [1]. EAU guidelines for high-risk upper urinary tract cancer after radical nephroureterectomy advise regular cystoscopy and urinary cytology at three months, followed by repeat cystoscopy and cytology every three months for two years, then every six months until five years, and yearly after that. The guidelines also advise performing a CT urogram and CT chest every six months for two years, then yearly, to monitor for potential recurrence or metastasis [1]. We are reporting this case of primary ureteral CIS due to its rarity, and to provide valuable learning points regarding the clinical significance of the condition, its association with other urological conditions, and the optimal management strategies. This knowledge contributes to understanding the disease and helps guide clinical practice, ultimately improving patient outcomes.

## Conclusions

A multimodal diagnostic approach, including ureteroscopy, cytology, and imaging, is essential for accurately diagnosing UT-CIS. Additional techniques such as PDD or NBI can enhance the detection of CIS during ureteroscopy. Treatment options for UT-CIS include radical nephroureterectomy and BCG therapy, with the choice depending on individual patient factors and limited evidence supporting the use of endocavitary treatments in UT-CIS. Further research and validation of diagnostic and treatment modalities are needed to optimize the management of UT-CIS.
